# Eyes on Lipinski's Rule of Five: A New “Rule of Thumb” for Physicochemical Design Space of Ophthalmic Drugs

**DOI:** 10.1089/jop.2021.0069

**Published:** 2022-01-28

**Authors:** Thomas K. Karami, Shumet Hailu, Shaoxin Feng, Richard Graham, Hovhannes J. Gukasyan

**Affiliations:** Pharmaceutical Sciences, Allergan plc, an AbbVie Company, Irvine, California, USA.

**Keywords:** ophthalmic drug delivery, physicochemical design space, formulation, solubility, ocular permeability

## Abstract

The study objective was to investigate molecular thermodynamic properties of approved ophthalmic drugs and derive a framework outlining physicochemical design space for product development. Unlike the methodology used to obtain molecular descriptors for assessment of drug-like properties by Lipinski's Rule of 5 (Ro5), this work presents a retrospective approach based on *in silico* analysis of molecular thermodynamic properties beyond Ro5 parameters (ie, free energy of distribution/partitioning in octanol/water, dynamic polar surface area, distribution coefficient, and solubility at physiological pH) by using 145 marketed ophthalmic drugs. The study's focus was to delineate inherent molecular parameters explicitly important for ocular permeability and absorption from topical eye drops. A comprehensive parameter distribution analysis on ophthalmic drugs’ molecular properties was performed. Frequencies in distribution analyses provided groundwork for physicochemical parameter limits of molecular thermodynamic properties having impact on corneal permeability and topical ophthalmic drug delivery. These parameters included free energy of partitioning (ΔG_o/w_) calculated based on thermodynamic free energy equation, distribution coefficient at physiological pH (*c*log *D*_pH7.4_), topological polar surface area (TPSA), and aqueous solubility (*S*_int_, *S*_pH7.4_) with boundaries of *c*log *D*_pH7.4_ ≤4.0, TPSA ≤250 Å^2^, ΔG_o/w_ ≤20 kJ/mol (4.8 kcal/mol), and solubility (*S*_int_ and *S*_pH7.4_) ≥1 μM, respectively. The theoretical free energy of partitioning model streamlined calculation of changes in the free energy of partitioning, Δ(ΔG_o/w_), as a measure of incremental improvements in corneal permeability for congeneric series. The above parameter limits are proposed as “rules of thumb” for topical ophthalmic drugs to assess risks in developability.

## Introduction

The *in vivo* biopharmaceutical assessment of topical ophthalmic drugs is challenging due to practical difficulties with tissue sampling during clinical studies.^1^ This creates a need for *in vitro* permeability assessment combined with *in silico* predictions and modeling as practical approaches for molecular property determination and biopharmaceutic predictions for developability assessment of new ophthalmic drug candidates. A general “rule of thumb” for valuation of drug-like properties, known as Lipinski's rule of 5 (Ro5), has been introduced for almost 2 decades,^2^ which is a generally accepted method to predict drugs’ ADME (“absorption, distribution, metabolism, and excretion”) performance mainly for oral drugs. To develop the Ro5, Lipinski analyzed 2,245 compounds at the entry to Phase II of development programs, retrospectively.

Lipinski identified which physicochemical properties are common within the selected compounds.^3^ Analogously, Choy and Prausnitz^4^ studied a total of 111 drugs approved for 3 nonoral routes of administration, of which 59 were ophthalmic, 39 were inhalation, and 17 were transdermal drugs. They found that >98% of the limited selection of ophthalmic drugs (59 drugs) possess molecular descriptors within the boundaries outlined by Ro5. They concluded that, although ophthalmic drugs follow the Ro5, these guidelines should not be loosely applied to assess developability of other parenteral drug candidates, especially those for inhalation and transdermal delivery. Their dataset contained drugs with descriptors outside the Ro5 limits, for example, several hydrophilic macromolecules absorbed by inhalation and transdermal drugs that fall within stricter limits than prescribed by Ro5. Since the Choy and Prausnitz^4^ evaluation addressed <50% of known topical ophthalmic drugs on the market in 2010–2011, possibly introducing bias in resulted aggregate compliance, they may have also artificially limited exploration of any other physicochemical parameter outside the scope of Ro5.

Shirasaki,^5^ in a comprehensive review, discussed a trend with ophthalmic drugs being adopted from systemic drugs, while the status quo necessitates molecular design for new drug candidates for treatment of ocular diseases. Relying largely on evidence obtained from published nonclinical ocular pharmacokinetic studies, Shirasaki proposed a pragmatic approach for molecular design to obtain optimum ocular permeability for topical delivery to the eye.^5^

As a fundamentally different approach than the reviews by Choy and Prausnitz,^4^ and Shirasaki,^5^ this study is designed to investigate the following: (1) distribution of physicochemical parameters beyond the Lipinski's Ro5 [ie, topological polar surface area (TPSA), calculated distribution coefficient at physiological pH (*c*log *D*_pH7.4_), molecular free energy of distribution/partitioning (ΔG_o/w_), and calculated intrinsic solubility (*S*_int_)/solubility at physiological pH (*S*_pH7.4_)] by taking into account all approved ophthalmic drugs to obtain a physicochemical design space for ophthalmic drug delivery; and (2) correlations between the outlined design space parameters and *in vitro* ocular permeability (corneal and conjunctival) reported in the literature. Overall, the main objective of this study was to outline a “physicochemical design space” that aims to emphasize molecular parameters relevant to topical ophthalmic absorption, which can potentially be used for developability assessment of new ophthalmic drug candidates.

Approved ophthalmic drugs (*n* = 145) used for this study are currently listed as active pharmaceutical ingredients in ophthalmic products based on the U.S. Food and Drug Administration's Orange Book and Drug Bank,^6,7^ which are databases accessible to the public. Frequency and distribution of molecular thermodynamic parameters, that is, TPSA, *c*log *D*_pH7.4_, ΔG_o/w_, and solubility, were calculated to demarcate and define their boundaries. To evaluate correlation between the above-mentioned design space parameters and corneal permeability, a subset of 42 ophthalmic drugs with accessible experimental permeability data reported in literature (corneal and conjunctival permeability in rabbit^1^ and porcine^8^) were used.

## Physicochemical Design Space and “Rule of Thumb for Ophthalmic Drugs” (RO_x_)

Physicochemical characteristics of drugs are critical parameters for ophthalmic drug delivery.^9^ Cornea is considered primary route of drug penetration into anterior segment from topical eye drops. Since it is a multilayered tissue, the rate-limiting step of corneal permeation is drug-dependent, which relates to the physicochemical properties of drugs. Partially due to challenges with permeation of drug through the cornea and anterior eye tissues, the intraocular bioavailability of the topically administered drugs ends up being low,^1^ ranging from 5% to 10%. Ahmed and Patton introduced a system that allowed an *in vivo* examination of noncorneal absorption of drugs to the intraocular space by topical dosing to albino rabbits.^10^ They used timolol and inulin as the probe drugs. The results of their study mechanistically illustrated the role of noncorneal pathway for absorption into intraocular tissues. Inulin with a high molecular weight (MW), impermeable through cornea, was shown to penetrate the intraocular space through the noncorneal pathway, primarily conjunctiva.^10,11^

Kidron et al.^12^ developed a computational model for prediction of corneal permeability by using multivariate analysis based on molecular descriptors [eg, log *P*, log *D*_pH7.4_, nHBA (number of H-bond acceptors), nHBD (number of H-bond donors), nHB_tot_ (total number of H-bonds), polar surface area, molecular volume, and MW] of drug-like compounds. In the first study, effect of physicochemical factors such as MW, distribution coefficient (log *D*), pKa, and degree of ionization on corneal permeability was investigated. The corneal permeability values were measured by modified perfusion chambers. Several correlations between the “log of permeability coefficient” (log *P*_coeff._) versus sum of “log-functions” of partition coefficient (log *D*), MW, and degree of ionization were examined. The correlation between log *D*_pH7.4_ and corneal permeability was also later studied extensively by Kidron et al. and confirmed.^12^

Permeation of drugs across the cornea was shown to increase with lipophilicity of beta-blockers following a sigmoidal relationship, which was shown to be in good agreement with the corneal permeability of beta-blockers as reported by Huang and Schoenwald.^13^ The ratio of corneal to conjunctival permeability coefficient was shown to be mostly sensitive to changes in the partition coefficient (log *D*) of drugs at pH 7.4 within the range of log *D* from 1.5 to 2.5.^8,14^ Schoenwald and colleagues’ investigations were also focused on corneal permeability of beta-blocking drugs, which were reported in 3 successive publications.^13,15,16^

As it was shown by Patton and colleagues,^10,11^ besides the cornea, the potential route for ocular absorption is paracellular penetration of drugs across conjunctiva and sclera. Sclera has shown to be the main path for absorption of both high- and low-MW compounds, for example, inulin (C_6n_ H_10n + 2_ O_5n + 1_, *n* = 2–60) and p-aminoclonidine (MW = 245.11 g/mol). Prausnitz and Noonan^17^ studied empirical correlations between corneal and conjunctival permeability and molecular descriptors such as MW, Van der Waals radii, partition coefficients (log *P* and *c*log *P* values), distribution coefficient (log *D*), and ionized fraction of drug at physiological pH.

The previously mentioned literature provides a background about the importance of understanding the impact of molecular properties of drugs on ocular permeability and absorption. Except for the distribution coefficient at ocular pH range, other molecular parameters that we focused on in this study [ie, TPSA and free energy of partitioning (ΔG_o/w_) across corneal or conjunctival tissues], are novel parameters to be considered for physicochemical design space of ophthalmic drugs. TPSA is known to have impact on biological cell absorption as reported in the literature.^18,19^ In addition to the TPSA and ΔG_o/w_ parameters, we studied distribution coefficient (log *D*) and solubility at pH 7.4 as 2 measurable composite parameters that have been emphasized as important properties for ophthalmic drugs.^20^ A special emphasis will be given to the relationships between these parameters in the physicochemical design space for RO_x_ and the *in vitro* permeability of ophthalmic drugs through corneal and conjunctival tissues reported in the literature.^1,8^

**FIG. 1.**
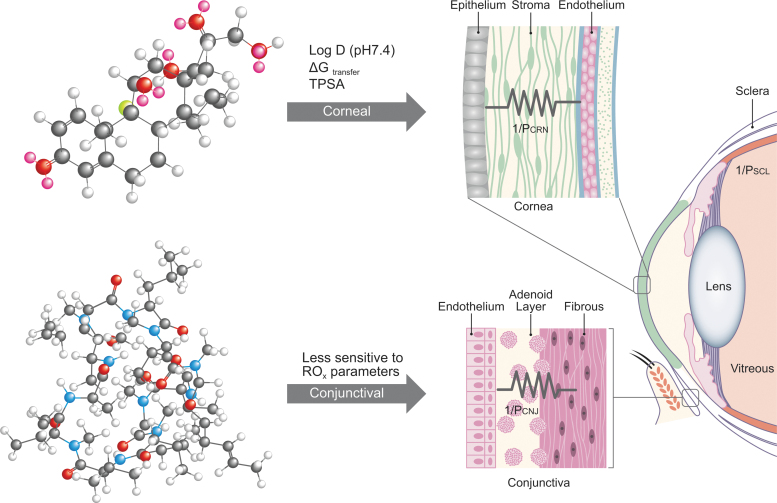
Composite physicochemical parameters in RO_x_ are listed over *arrows* depicting 2 possible absorption routes into the eye following topical dosing. The corneal and conjunctival tissue barriers are shown graphically with overlay symbols of parallel resistors (eg, their combined simultaneous conductance, or total drug flux, can be modeled as sum of corneal and conjunctival permeability values, *P*_CRN_ + *P*_CNJ_, respectively). While *P*_CRN_ is sensitive to RO_x_, *P*_CNJ_ displays lower sensitivity playing an increasingly important role in ocular exposure of compounds with poor intrinsic corneal penetration. *In vitro* permeability data in rabbit and porcine cornea and conjunctiva suggest that corneal permeability is impacted by the *c*log *D*_pH7.4_ (distribution coefficient at physiological pH), TPSA, and ΔG (free energy of partitioning/transfer)—*top* ball-and-stick model drug dexamethasone proposed preferential route of absorption, while the conjunctival permeability is less sensitive to the parameters in RO_x_—*bottom* ball-and-stick model drug cyclosporin A proposed preferential route of absorption (c.f. data in Tables 2 and 3 and the Results and Discussion section). TPSA, topological polar surface area.

Figure 1 is a graphical summary of proposed impact of the physicochemical parameters in RO_x_ on drug permeability through biological barriers of the eye. Appropriate contextual considerations include precorneal physiological and biophysical barriers presented by the eye, such as a drug-surface concentration profile limited by significant dilution in resident tear film, blink reflex, and rapid drainage to maintain constant tear film volume, to drug-like molecules’ absorption across 2 primary membranes.^1^ As a result, the model shown by Fig. 1 relies on rapidly changing, nonsteady-state partitioning kinetics, and flux of drugs through corneal tissues impacted by parameters in RO_x_ (ΔG_o/w_, TPSA, and log *D*_pH7.4_). Transport across corneal epithelium (a lipophilic layer), stroma (hydrophilic layer containing collagen fibers), and endothelium (another lipophilic monolayer) occurs by both transcellular and paracellular mechanisms (upper steroidal model drug, dexamethasone acetate; Fig. 1). On the other hand, permeability of drugs through the vascularized, relatively hydrophilic conjunctival tissue consisting of epithelial, adenoid, and fibrous layers is less sensitive to the RO_x_ parameters as the primary mechanism for absorption is dominated by paracellular pathway (lower cyclic peptide model drug, cyclosporine; Fig. 1). Absorption across sclera involves passive diffusion through perivascular pore pathways with least resistance to the drug-like molecule transport. Notably, *in vivo* conditions would offer both routes (eg, corneal and conjunctival) of absorption to a drug-like molecule from an instilled eye drop, simultaneously and in parallel, and based on the compounds’ intrinsic RO_x_ parameters, absorption will occur through the path of least resistance [depicted by parallel resistor symbols as the sum of 1/*R*_app_ cornea (1/R_crn_), 1/*R*_app_ conjunctiva (1/R_cnj_), and 1/*R*_app_ sclera (1/R_scl_), where *R* represents tissue resistance; Fig. 1]. In this study, the apparent experimental *in vitro* permeability (*P*_app_) for 42 ophthalmic drugs through corneal and conjunctival tissues of rabbit and porcine was examined for correlation with RO_x_ parameters, which will be discussed in the Results and Discussion section and shown in Supplementary Table S1.

### Log *D* at tear film pH (7.4)

**FIG. 2.**
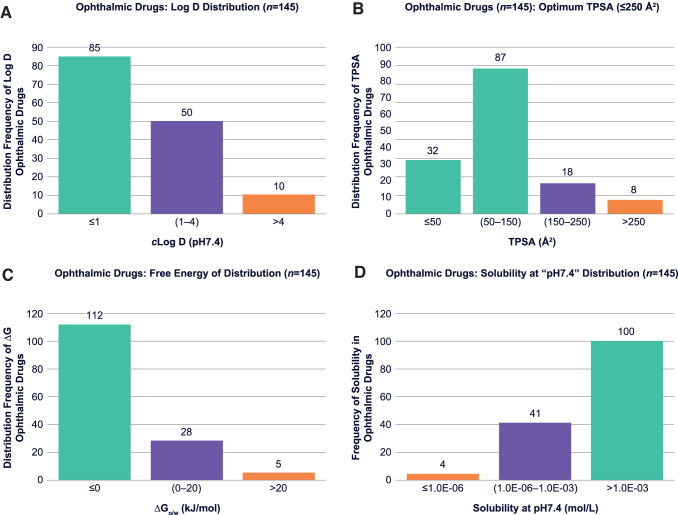
Histograms on frequency of distribution of RO_x_ parameters; *c*log *D*_pH7.4_ **(A)**, TPSA **(B)**, ΔG_o/w_ **(C)**, and *S*_pH7.4_ **(D)**, within the approved ophthalmic drugs. The *orange* bars present number of ophthalmic drugs that are outliers. The *green* and *purple bars* represent number of drugs that outline parameter criteria for RO_x_: i.e., *c*log *D*_pH7.4_ ≤4.0 **(A)**, TPSA ≤250 Å^2^ **(B)**, *Δ*G_o/w_ ≤20 kJ/mol **(C)**, and *S*_pH7.4_ ≥1 μM **(D)**. *c*log *D*_pH7.4_, distribution coefficient at physiological pH; ΔG_o/w_, free energy of partitioning/distribution; ROx, “rule of thumb for ophthalmics”; *S*_pH7.4_, solubility at physiological pH.

The impact of log *D* on ocular permeability has been described in the literature as stated earlier. Prausnitz and Noonan^17^ emphasized that corneal permeability appears to be function of distribution coefficient with a trend showing permeability increases upon increase of log *D* (pH 7.0–7.65). However, the frequency of distribution coefficient at pH 7.4 within the approved ophthalmic drugs has not been reported. Our data analysis on distribution and frequency of *c*log *D*_pH7.4_ values (Fig. 2A) indicates that the majority of approved ophthalmic drugs have *c*log *D*_pH7.4_ ≤4.0 (RO_x_, Rule #1).

For nonionizable drugs, the *c*log *D*_pH7.4_ values are the same as *c*log *P*. We obtained the *c*log *D* and *c*log *P* values by *in silico* calculations using ACD Percepta (2017.1.1; ACD Labs, Ontario, Canada),^21^ which will be described in the Experimental Methods and *In Silico* Predictions and Results and Discussion sections. The parameter distribution data (Fig. 2A) suggested that ophthalmic drugs have a wide range of distribution coefficient at pH 7.4 from highly hydrophilic drugs (*c*log *D* ≤ 1) to fairly lipophilic (*c*log *D* = 1–4).

### Topological polar surface area

The TPSA of drug molecules has been reported to have a direct impact on drug absorption across the biological cell membranes such as Caco-2 (large intestine carcinoma cells),^18^ brain, and nerve cells in the central nervous system.^19^ These studies reported that drugs with dynamic TPSA <60 Å^2^ are completely absorbed, whereas those with TPSA >140 Å^2^ will have restricted permeation.^18,19^ The TPSA data for approved ophthalmic drugs analyzed in this study indicated that the majority of the ophthalmic drugs have TPSA ≤150 Å^2^, which is in good agreement with the optimum TPSA range (60–140 Å^2^) reported for cellular absorption.

Analysis on distribution frequency of ophthalmic drugs versus TPSA shows that a vast majority of ophthalmic drugs have TPSA ≤250 Å^2^ (RO_x_, Rule #2; Fig. 2B). Those drugs that have TPSA values at 150–250 Å^2^ are mainly antibacterial, anti-inflammatory, or secretagogues used to treat dry eye conditions, which do not necessitate ocular absorption for efficacy. The TPSA values for all 145 approved ophthalmic drugs were predicted by the ACD Percepta software. The data set and result of the parameter distribution analysis on TPSA within the ophthalmic drugs will be discussed methodically in the Results and Discussion section.

### ΔG_o/w_ (free energy of distribution/partitioning)

The biophysical basis for ocular membrane permeability is well known, as described in the literature, but theoretical models to predict free energy of distribution for drug molecules across epithelial membranes of the eye (eg, cornea and conjunctiva) have not been explored. The free energy of distribution values for approved ophthalmic drugs was calculated using theoretical equations adapted from the literature such as Anderson et al.^22^ and Leung et al.^23^ While this is analogous to the partition coefficient in oil/water, that is, the Lipinski RO5 composite parameter, it differs by means of allowing for a semiquantitative prediction of preferential accumulation and passage through or between epithelial cells comprising the entire ocular surface (ie, cornea and conjunctiva).

To calculate changes in free energy of partitioning “Δ(ΔG)” for congeneric series of incrementally modified versus initial drugs described by Shirasaki,^5^ we used the partition coefficient values (*c*log *P*) for unionized drug and distribution coefficient (*c*log *D*_pH7.4_) for ionized drug. The thermodynamic free energy equations used for prediction of Δ(ΔG) and ΔG_o/w_ will be described in detail in the Results and Discussion section. Analysis of distribution frequency of ophthalmic drugs versus ΔG_o/w_ (Fig. 2C) confirms that the vast majority of ophthalmic drugs have ΔG_o/w_ ≤20 kJ/mol, which is proposed as the parameter limit (RO_x_, Rule #3).

### Solubility (intrinsic vs. tear film pH)

Solubility (eg, *S*_int_) is an important physicochemical parameter that has impact on both topical ophthalmic drug delivery and formulation development. For example, if a dose/solubility ratio is ≥1 for a putative drug-like molecule, solubilization becomes limiting for topical ophthalmic formulation development. The net charge and solubility of ionizable compounds displaying pH-dependent behavior are most relevant at physiological tear film pH. Ideally, the pH of ophthalmic eye drops should be equivalent to that of tear fluid, approximately pH 7.4.

**FIG. 3.**
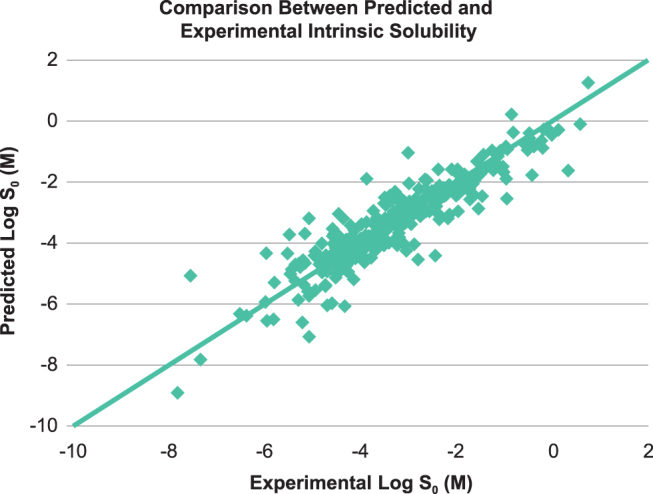
Comparison between predicted and experimental intrinsic solubility (log *S*_0_) of 289 commercial drugs (including ophthalmic and oral drugs).

In this study, due to limited experimental solubility data reported for all 145 ophthalmic drugs, solubilities were calculated (both *S*_int_ and *S*_pH7.4_). Values obtained by ACD Percepta were then compared with the available experimental solubility data for a larger population of commercial drugs (Fig. 3), including ophthalmic drugs, to obtain a qualitative comparison of theoretical versus experimental results (see the Results and Discussion section). Distribution analysis for solubility data in approved ophthalmic drugs was performed on calculated values to show boundaries for this parameter, which appeared to be ≥1 μM (RO_x_, Rule #4) (Fig. 2D).

In summary, boundaries for RO_x_ outlined by parameter distribution analyses are as follows: RO_x_, Rule #1: *c*log *D*_pH7.4_ ≤4.0, RO_x_, Rule #2: TPSA ≤250Å^2^, RO_x_, Rule #3: ΔG_o/w_ ≤20 kJ/mol (4.8 kcal/mol), RO_x_, and Rule #4: Solubility (*S*_int_ and *S*_pH7.4_) ≥1 μM.

## Ocular Drug Absorption

### Corneal absorption

Among the outer epithelium, middle stroma, and inner endothelium, corneal epithelium is the lipophilic rate-limiting barrier containing 5–7 multilayers of epithelial cells with tight junctions.^17,24^ As a result, transcellular transport is the predominant mechanism of absorption through the corneal epithelium for lipophilic drugs, while paracellular transport governs hydrophilic molecules.^24^ A linear correlation reported for epithelial permeability versus log *D* indicated that drug lipophilicity is critical for absorption across the corneal epithelium.^15^ A sigmoidal relationship of corneal permeability versus lipophilicity is reported for various classes of drugs, with maximum permeation at optimum log *D* of ∼2–3.^13–15,24^ The stroma is a hydrophilic tissue containing mainly collagen fibers, noncollagenous proteins and glycosaminoglycans filled with water (∼78%), and some keratocytes.^15^ Drug transport across stroma involves passive diffusion through aqueous pore pathways and, thus, it is a rate-limiting layer for corneal absorption of lipophilic drugs that readily permeate through the corneal epithelium.^15^ The endothelium is a monolayer of cells joined through gap junctions. The dependence of endothelial permeability on log *D* and molecular size is reported, indicating absorption involves both transcellular and paracellular pathways.^17^ Consequently, molecular diffusion across endothelium occurs readily through paracellular and transcellular pathways, concluding transcorneal absorption.^15,17,24,25^ Taken on a global organ level, since the corneal epithelium plays a hydrophobic rate-limiting step and is continuous with the conjunctival epithelium (eg, the conjunctiva starts at the limbus where the cornea ends), large and/or hydrophilic molecules frequent paracellular diffusion through corneal and noncorneal putative water-filled pores.

### Conjunctival-scleral absorption

Conjunctival-scleral permeability, considered the noncorneal route, plays a complementary parallel role in ocular drug absorption. Conjunctival epithelial multilayer cells (5–15 layers) offer lower resistance to drug-like molecules versus corneal epithelium. They secrete mucin and have a unique vascularized stroma, unlike the completely avascular cornea. Drug absorption across conjunctiva occurs through transcellular and paracellular routes. Highly dependent on drugs’ physicochemical properties, the conjunctiva is reported to have similar or greater permeability than cornea,^11,14,17^ due to the 17 times larger surface area and lower transepithelial resistance.^20,26^ The higher permeability of conjunctiva than cornea reported for inulin demonstrated that conjunctiva is the likely route for ocular absorption of hydrophilic macromolecules.^11^ The sclera is a hydrophilic tissue containing ∼70% water, composed of collagens, noncollagenous proteins, glycosaminoglycans, and some fibroblast cells. Scleral permeability is in general higher than corneal and comparable with or higher than conjunctival permeability^11^; however, lower permeability of sclera than conjunctiva was reported for polyethylene glycol oligomers.^21^ As the anterior sclera is preceded by the bulbar conjunctiva, from the perspective of an instilled eye drop, drug absorption across sclera is ancillary and involves passive diffusion through aqueous media and perivascular pore pathways.^11,27^

### Carrier-mediated transport in ocular tissues

In addition to passive diffusion (transcellular and paracellular), several transporters are expressed in cornea, conjunctiva, and other eye tissues, which are suggested to be involved in carrier-mediated active uptake and efflux of substrates.^11,24,27^ While corneal absorption through active transport may be limited due to the short residence time after instillation or kinetic saturation of transporters by virtue of high initial concentration of topically applied ophthalmic drugs, drug-like molecules with any extent of interaction with active transport fall outside molecular thermodynamic property-related passive diffusion criteria. In fact, outliers (ie, acebutolol^13,16,17^ Tables 1 and 2) in RO_x_ warrant further analysis to understand lack of, or higher than, expected empirical observations in topical ophthalmic absorption.

**Table 1.** Summary of Ophthalmic Drugs That Deviate from Rule of Ophthalmic Drugs (RO_x_) and Lipinski's Rule of 5

Drug's name	Ophthalmic indication	Outliers from RO_x_ limits				Outliers from Ro5			
		clog *D*_pH7.4_	TPSA (Å^2^)	c *S*_pH7.4_ (M)	ΔG_o/w_ (kJ/mol)	MW (Da)	nHBD	nHBA	clog P
Acetylcholine chloride	Miotic agent	−3.50	26.3	6.0E-01	**20.77**	146.21	0	3	−3.5
Azithromycin	Antibacterial	0.15	180.1	1.0E+0	−19.53	**748.98**	5	**14**	3.29
Aztreonam	Antibacterial	−6.21	238.2	5.9E+0	7.18	435.44	5	**13**	−1.21
Bacitracin	Antibiotic	−5.77	**556.2**	1.0E-05	18.76	**1,422.7**	**20**	**33**	−3.16
Brilliant blue G	Ocular surgical stain	−0.38	153.0	**4.0E-08**	5.82	**833.05**	3	10	−0.98
Chlortetracycline	Antibacterial	−2.43	182.6	1.4E-03	−3.86	478.88	**7**	10	0.65
Cromolyn sodium	Antiallergy	−2.95	165.9	2.1E+0	−10.68	468.37	3	**11**	1.80
Cyclosporine	Dry eye agent	1.80	**278.8**	9.8E-06	−10.68	1,202.6	5	**23**	1.80
Demecarium	Esterase inhibitor	−3.14	59.1	1.1E-05	18.64	**556.78**	0	8	−3.14
Diquafosol tetrasodium	Dry eye agent	−15.48	**432.8**	7.1E+0	**50.33**	**790.31**	**10**	**27**	−8.48
Erythromycin	Antibacterial	1.69	193.9	1.3E-02	−14.48	**733.93**	5	**14**	2.44
Gentamicin	Antibacterial	−7.90	199.7	2.1E+0	12.76	477.60	**11**	**12**	−2.15
Gramicidin D	Antibacterial	**5.54**	**519.9**	**2.5E-12**	−32.88	**1,811.2**	**20**	**35**	**5.54**
Isopropyl unoprostone	Ocular hypertension	**4.79**	83.8	2.5E-05	−28.43	424.61	2	5	4.79
Latanoprost	Ocular hypertension	**4.11**	87.0	2.3E-04	−24.40	432.59	3	5	4.11
Latanoprostene bunod	Ocular hypertension	**4.25**	145.1	3.3E-05	−25.23	**507.62**	3	9	4.25
Lifitegrast	Conjunctivitis	−0.73	142.4	3.5E-04	−19.05	**615.48**	2	9	3.21
Liothyronine	Thyroid eye disease	**4.07**	92.8	2.9E-06	−24.45	**650.97**	4	5	4.12
Loratadine	Antiallergy	**5.32**	42.4	2.5E-06	−31.58	382.88	0	4	**5.32**
Methotrexate	Anti-inflammatory	−5.42	210.5	8.9E-02	3.32	454.44	**7**	**13**	−0.56
Natamycin	Antibacterial	−2.92	231.0	7.9E-03	2.37	**665.73**	**8**	**14**	−0.40
Neomycin	Antibacterial	−9.15	**353.1**	7.1E+0	**28.97**	**614.64**	**19**	**19**	−4.88
Oxytetracycline	Antibacterial	−4.25	201.9	1.3E-02	7.84	460.43	**8**	**11**	−1.32
Polymyxin B	Antibacterial	−11.16	**490.7**	8.3E-01	**25.05**	**1,203.5**	**23**	**29**	−4.22
Tacrolimus	Conjunctivitis	**4.10**	178.4	5.1E-06	−24.34	**804.02**	3	**13**	4.1
Tafluprost	Ocular hypertension	**4.24**	76.0	5.2E-05	−25.17	452.53	2	5	4.24
Tobramycin	Antibacterial	−7.22	**268.2**	7.1E+0	**24.51**	467.51	**15**	**14**	−4.13
Travoprost	Ocular hypertension	3.98	96.2	3.4E-05	−23.62	**500.55**	3	6	3.98
Trypan Blue	Ocular surgery stain	−9.56	**392.9**	1.5E-02	18.16	**872.88**	**10**	**20**	−3.06
Vitamin E	Ultraviolet protection	**10.3**	29.5	**8.3E-09**	−61.14	430.71	1	2	**10.3**
Vizomitin	Antioxidant	**4.23**	34.1	**5.6E-10**	−25.11	**537.69**	0	2	4.23

The molecular descriptors of the ophthalmic drugs that are outliers and deviate from parameters limit for RO_x_ and Ro5 are highlighted in bold.

ΔG_o/w_, free energy of partitioning; log *D*_pH7.4_, distribution coefficient at physiological pH; log *P*, partition coefficient; MW, molecular weight; nHBA, number of H-bond acceptors; nHBD, number of H-bond donors; Ro5, rule of 5; Ro5, Lipinski's Rule of 5; RO_x_, “rule of thumb” for ophthalmics; *S*_pH7.4_, solubility at physiological pH; TPSA, topological polar surface area.

**Table 2.** Summary of Corneal and Conjunctival Permeability in Rabbit^1^ Versus RO_x_ Parameters

Drug's name	Corneal permeability, *P*_app, CRN_ (cm/s)	Conjunctival permeability, *P*_app, CNJ_ (cm/s)	Molecular parameters in RO_x_		
			clog *D*_pH7.4_	TPSA (Å^2^)	ΔG_o/w_ (kJ/mol)
Acebutolol	3.62E-06	3.24E-06	−0.38	87.7	−9.972
Acetazolamide	1.28E-06	3.39E-06	−0.68	151.7	1.840
Apraclonidine	3.65E-06	1.26E-05	−0.41	62.4	−8.191
Atenolol	1.79E-06	4.95E-06	−1.82	84.6	−1.425
Betaxolol	3.65E-05	5.24E-06	0.81	50.7	−17.035
Brimonidine	2.88E-05	6.73E-06	−1.97	62.2	−5.639
Brinzolamide	9.10E-07	5.15E-06	−0.15	163.8	0.059
Bufuralol	2.24E-05	3.58E-06	1.42	45.4	−21.309
Ciprofloxacin	4.20E-07	4.84E-06	−2.6	72.9	1.781
Clonidine	4.67E-05	1.26E-05	1.18	36.4	−10.684
Dexamethasone	5.08E-06	4.38E-06	1.92	94.8	−11.396
Dexamethasone acetate	1.95E-05	5.44E-06	2.82	100.9	−16.738
Dorzolamide	9.90E-07	4.17E-06	−1.19	151.3	1.306
Ethoxzolamide	2.59E-05	1.90E-06	1.75	118.9	−10.922
Fluorescein	1.07E-06	3.84E-06	3.55	76.0	−21.131
Latanoprost	9.68E-05	4.77E-06	4.11	87.0	−24.395
Latanoprost acid	5.90E-07	2.59E-06	0.26	98.0	−16.323
Levobunolol	1.95E-05	5.51E-06	0.44	58.6	−15.255
Moxifloxacin	8.91E-06	5.98E-06	−1.47	82.1	−4.630
Propranolol	3.80E-05	2.48E-06	1.2	41.5	−19.350
Testosterone	3.29E-05	2.20E-06	3.16	37.3	−18.757
Timolol	1.89E-05	5.15E-06	−0.79	108.0	−9.081

*P*_app, CRN_, corneal permeability; *P*_app, CNJ_, conjunctival permeability.

**Table 3.** Summary of Corneal and Conjunctival Permeability in Porcine^8^ Versus RO_x_ Parameters

Drug's name	Corneal permeability, *P*_app, CRN_ (cm/s)	Conjunctival permeability,* P*_app, CNJ_ (cm/s)	Molecular parameters in RO_x_		
			clog* D*_pH7.4_	TPSA (Å^2^)	ΔG_o/w_ (kJ/mol)
Aciclovir	7.29E-06	2.03E-06	−1.23	114.8	7.301
Ampicillin	1.89E-07	1.35E-06	−1.86	138.0	−5.579
Atropine	5.64E-07	4.37E-06	−0.35	49.8	−11.040
Bromfenac	3.99E-07	4.02E-06	−0.1	80.4	−18.222
Carteolol	1.43E-07	2.75E-06	−0.3	70.6	−10.922
Diclofenac	6.20E-07	8.77E-06	1.47	49.3	−26.592
Fluconazole	9.97E-07	4.50E-06	0.7	81.7	−4.155
Ganciclovir	3.82E-06	1.88E-06	−1.72	135.0	10.209
Indomethacin	5.03E-07	6.23E-06	1.14	68.5	−23.861
Ketorolac	3.49E-07	1.79E-06	−0.92	59.3	−15.314
Levocabastine	4.08E-07	4.06E-06	1.98	64.3	−26.592
Lincomycin	9.00E-08	1.16E-06	0.27	147.8	−3.739
Methazolamide	4.74E-07	2.73E-06	−0.03	138.9	−1.899
Nadolol	1.22E-07	1.25E-06	−0.9	82.0	−7.360
Pilocarpine	1.79E-06	7.73E-06	0.23	44.1	-2.315
Pindolol	7.54E-07	5.72E-06	−0.2	57.3	−11.040
Prednisolone acetate	2.03E-07	2.73E-06	2.33	100.9	−13.830
Quinidine	8.68E-07	4.12E-06	1.82	45.6	−17.926
Tizanidine	4.70E-06	9.90E-06	−1.63	90.4	−7.657
Voriconazole	1.73E-06	7.55E-06	1.39	76.7	−8.251

*P*_app, CRN_, corneal permeability; *P*_app, CNJ_, conjunctival permeability.

## Experimental Methods and *In Silico* Predictions

The drug substances selected for physicochemical property evaluation were approved, compendial drugs for ophthalmic indications (Supplementary Table S1). The molecular parameters in RO_x_ (*c*log *D*, TPSA, and *S*_pH7.4_) and descriptors in Lipinski's Ro5 (MW, log *P*, nHBA, and nHBD) were calculated for 145 ophthalmic drugs *in silico* by using ACD Percepta software, PhysChem ADMET (2017.1.1; ACD Labs). The corneal and conjunctival permeability (*P*_app_) values used in this study were obtained from the literature.^1,8^

### Determination of molecular descriptors

Ophthalmic drugs selected in this study were derived from a search in the U.S. Food and Drug Administration's Orange Book and Drug Bank databases.^6,7^ The ophthalmic drugs have been approved and marketed for ocular disease indications such as eye infection, ocular inflammation, dry eye syndromes, ocular hypertension, conjunctivitis, and glaucoma. The chemical structures of all ophthalmic drugs were obtained from Drug Bank,^7^ and then verified by ChemIDplus database.^28^ After confirmation, structures were used as inputs for *in silico* calculations of physicochemical descriptors with ACD Percepta. The data were then compiled in Excel for parameter distribution analysis by histogram plots (MS Office; Microsoft Corporation). The histogram method provides visual representation of distributions for parameters in RO_x_ and molecular descriptors in Lipinski's Ro5 based on the frequencies for each parameter, binning them in respective ranges, described by Karl Pearson's method.^29^ The distribution of a given target molecular descriptor within ophthalmic drugs is defined as “relative number of occurrences,” within the examined population.

### Determination of solubility

The calculated *S*_int_ and *S*_pH7.4_ for 145 ophthalmic drugs (Supplementary Table S1) were obtained by ACD Percepta. The software calculates pH-dependent and intrinsic aqueous solubility of molecules (unbuffered) at 25°C and zero ionic strength, along with the predicted equilibrium pH of the solution using Henderson-Hasselbalch theory for relationship between solubility, pKa, and pH. Acid dissociation constants (pKa) are calculated by Hammett-type equations for ionizable functional groups using derived electronic substituent constants (σ). A database of >17,000 compounds, representing >32,000 pKa values, is used in the classic algorithm module of ACD Percepta for determination of pKa.^21^ Reported experimental solubility data were all obtained from literature.^14,30^

### Prediction of differences in free energy of distribution Δ*(*Δ*G)*

To calculate differences in free energy changes, Δ(ΔG), for congeneric series of molecules reported in literature,^5^ the following equation [Eq. (1)] was used. The free energy of partitioning model considers molecular heterogeneity effect on the permeability versus partition coefficient relationships in tissues, for example, stratum corneum of human skin for transdermal drug delivery or corneal tissues for ophthalmic drug delivery, which will be applied here as follows:

(1) $$\Delta \left(\Delta G\right)=-2.303RT\times log\left({K_{R}}_{x}∕{K_{R}}_{H}\right)$$

where *K*_Rx_ and *K*_RH_ represent “permeability coefficients” in the biologic membrane for prodrug and the original drug molecule, respectively. Permeability coefficients are proportional to partition coefficients (log *P*), diffusivity, and membrane thickness. *R* is the gas constant (8.314 J/K mol) and *T* is the body's temperature (37°C, or 310°K).^22^

Anderson et al.^22^ introduced the above physicochemical model for prediction of changes in tissue permeability for congeneric series of drugs by calculation of changes in free energy of partitioning Δ(ΔG) upon modification of a reference drug molecule, for example, alkyl/aryl esters of an acidic drug. Equation (1) describes molecular thermodynamic Gibbs free energy at equilibrium by using partition coefficients, which was applied here to predict changes in free energy of partitioning (ΔG_o/w_) for ophthalmic congeneric series reported by Shirasaki.^5^ The results of the Δ(ΔG) predictions will be discussed in the Results and Discussion section.

### Prediction of free energy of distribution (ΔG_o/w_)

Free energies of distribution values were calculated by using Equation (2) adapted from Leung et al.,^23^ who applied the model introduced by Anderson et al.^22^ to assess passive permeation of drugs in biological membranes. Leung et al.^23^ used Equation (2), where they utilized partition coefficient of drugs in chloroform/water (log *P*_c/w_). Equation (1) was initially derived from Equation (2) by Anderson et al. to predict the free energy of partitioning/transfer based on the octanol/water partition coefficient (log P_o/w_):

(2) $$\Delta G_{o∕w}=logP_{o∕w}\left(-2.303RT\right)$$

where log *P* is the partition coefficient for the drug, *R* (8.314 J/K mol) is the gas constant, and *T* is the body temperature (37°C, or 310°K), as described earlier.^23^

Predicted free energy of distribution/partitioning values for the dataset describing approved ophthalmic drugs is shown in Supplementary Table S1, obtained by using calculated partition coefficients (*c*log *P*) for nonionizable drugs and *c*log *D*_pH7.4_ for ionizable drugs. Correlations between the free energy of partitioning versus *in vitro* permeability in rabbit and porcine corneal and conjunctival tissues were also studied, which appear in the Results and Discussion section.

## Results and Discussion

The molecular descriptors and physicochemical properties of all ophthalmic drugs listed in Supplementary Table S1 were examined to outline limits for RO_x_ (TPSA, *c*log *D*_pH7.4_, ΔG_o/w_, and *S*_pH7.4_) and look at compliance with Lipinski's Ro5 (MW, *c*log *P*, nHBD, and nHBA), adapting methods used by Lipinski,^2,3^ and Choy and Prausnitz.^4^ Special focus was on outlining the parameters’ limits for the RO_x_. Molecular property distributions of successfully developed ophthalmic drugs (Supplementary Table S1) were binned into subgroups within the parameter limits.

Based on Lipinski's method, if the analyzed drug substance candidates exceeded the boundary limits with >2 descriptors within the Ro5, they were classified as “ALERT 1.” The compounds that were identified with “ALERT 1” were considered “poor drug candidates” for oral administration. The development of such a drug was then evaluated as “at risk” or “challenging to develop,” which may require significant mitigation efforts.^4^ Current examination of molecular descriptors only includes drugs that have already passed development requirements and been approved for ophthalmic indications. As opposed to the term “ALERT,” herein instead “deviation” from parameter boundaries in “rule of thumb” for RO_x_ is used. Table 1 presents a summary of drugs with properties that deviate from descriptors within the RO_x_ and Ro5 highlighted in bold.

The histograms in Fig. 2A–D derived from the Supplementary Table S1 show parameter distribution analyses. Results indicate that only 19 drugs (∼13.1%) from the entire commercialized ophthalmic list deviate from RO_x_, of which 12 drugs deviate by 1 descriptor and 6 drugs deviate by 2 descriptors (tacrolimus, tobramycin, azithromycin, cyclosporine, methotrexate, and oxytetracycline). The only ophthalmic drug that breaks 3 rules within the RO_x_ is a high-MW antibiotic (Gramicidin D), which notably deviates with all 4 parameter criteria in Ro5. A close look at all 19 outliers from RO_x_ indicates that majority of these compounds are antibacterial, anti-inflammatory, miotic, and dry eye syndrome agents, which may not require complete ocular absorption for efficacy. Nevertheless, if we exclude the ophthalmic drugs that are antibacterial, anti-inflammatory, miotic, and dry eye agents from the outliers, 96% of the commercialized ophthalmic drugs have molecular parameters within suggested boundary limits of RO_x_.

Distribution of *c*log *D* at pH 7.4 shows that the majority of ophthalmic drugs have *c*log *D* ≤ 4.0 (Fig. 2A). The data also suggest that ophthalmic compounds have a wide range of distribution coefficients, from hydrophilic (*n* = 85) drugs with *c*log *D* ≤ 1 to lipophilic (eg, 50 drugs with *c*log *D* = 1–4). The largest ophthalmic product group has been developed with compounds that have *c*log *D* ≤ 4.0 (93.1%). Several compounds with extreme lipophilicity or *c*log *D* ≥ 4 have been also developed as topical ophthalmic products (eg, liothyronine tacrolimus, latanoprostene bunod, tafluprost, latanoprost, and loratadine). In topical ophthalmic product development, the pharmacological mechanism and site of action, as well as compounds’ potency to total dose relationship, need to be considered for appropriate eye drop formulation development. Therefore, based on highest frequency of appearance, a parameter limit for *c*log *D* for successfully developed ophthalmic drugs is ≤4.0 (RO_x_, Rule #1). If *c*log *D* ≥ 4, additional target product attributes for appropriate product vehicle development would be required.

The parameter distribution analysis on TPSA for approved ophthalmic drugs indicates that 82.1% of commercialized ophthalmic drugs have TPSA ≤150 Å^2^, which are in good compliance with the optimum TPSA range reported for the cell penetration.^18,19^ Fig. 2B displays frequency of ophthalmic drugs versus TPSA, confirming that a total of 94.5% of ophthalmic drugs have TPSA ≤250 Å^2^, of which 8 drugs (5.5%) have TPSA values >250 Å^2^ (see orange bar in Fig. 2B). Similar to *c*log *D*, these drugs that deviate from RO_x_ boundary are mainly antibacterial, anti-inflammatory, or dry eye agents (compare Fig. 2B with Table 1), which do not necessarily require ocular absorption for efficacy. Therefore, a limit for TPSA as a parameter is proposed at ≤250 Å^2^ (RO_x_, Rule #2). Furthermore, ophthalmic drug candidates that require cellular penetration to exhibit pharmacologic effect, for example, by permeation beyond ocular epithelial tissues to reach the iris ciliary body, aqueous humor or trabecular meshwork, the TPSA should be <150 Å^2^. Conversely, any ophthalmic drug candidate with a dynamic TPSA <50 Å^2^ should be expected to have limited restrictions for ocular absorption.

Calculated, using Equation (2), free energy of distribution/partitioning (ΔG_o/w_) was analyzed by parameter distribution analysis to outline the free energy limits. Based on the thermodynamic rule of free energy, if values of ΔG_o/w_ (free energy of distribution here) appear to be negative (ΔG_o/w_ <0), membrane absorption is expected to occur spontaneously. Figure 2C indicates 112 of 145 commercial ophthalmic drugs (77.2%) are theoretically capable of tear fluid-to-membrane partitioning without energetic restrictions at 35°C–37°C (eg, body or ocular surface temperature, 308°K–310°K) because of a negative free energy of transfer (ΔG_o/w_ <0). The orange bar in Fig. 2C captures acetylcholine chloride, diquafosol tetrasodium, neomycin, polymyxin B, and tobramycin, 5 hydrophilic (*c*log *P* ≤ −5) drugs, which are predicted to have a net-positive free energy of transfer ≥20 kJ/mol (4.8 kcal/mol). These molecules are also identified as antibacterial, dry eye, and miotic agents that do not require full absorption across the ocular tissues. Based on ΔG_o/w_ distribution analyses, 96.6% of approved ophthalmic drugs demonstrate a parameter limit for free energy of transfer at ΔG_o/w_ <20 kJ/mol (<4.8 kcal/mol), which is proposed as the RO_x_, Rule #3.

Parameter distribution analysis on the predicted solubility of commercial ophthalmic drugs at physiological, tear film pH (*S*_pH7.4_) is shown in Fig. 2D, indicating that the majority of molecules exhibits aqueous solubility at pH 7.4 > 1 μM. Therefore, the final proposed parameter limit for solubility is *S*_pH7.4_ ≥1 μM (RO_x_, Rule #4). If *S*_pH7.4_ ≤1 μM, solubilization by formulation technology may be required; however, it should be taken within context of Rules 1 through 3. Furthermore, 4 molecules (brilliant blue G-250, gramicidin D, vitamin E, and brand Visomitin) within the list of approved ophthalmic drugs have a calculated solubility ≤1 μM at pH 7.4. To verify, a search for their experimental aqueous solubilities indicated that there may be large experimental discrepancies. Calculated low-bin solubility for these 4 drugs is likely due to chemical structures being outside the prediction module training set of ACD Percepta.

The overall results of parameter distribution analyses for compliance with Lipinski's Ro5 (Table 1) also indicated that 27 ophthalmic drugs (18.6%) deviate from Ro5. Twelve of these drugs deviate with only 1 descriptor, 7 drugs deviate with 2 descriptors (tacrolimus, tobramycin, azithromycin, cyclosporine, methotrexate, and oxytetracycline), and 7 other drugs (bacitracin, diquafosol tetrasodium, neomycin, natamycin, polymyxin B, and trypan blue) deviate with 3 descriptors. Gramicidin D, a large MW antibacterial drug, breaks all 4 rules of Ro5 and deviates from RO_x_ by 3 parameters.^7^ Nonetheless, if we exclude antibacterial, anti-inflammatory, miotic, and dry eye agents from the outliers, 96% of commercialized ophthalmic drugs follow the Ro5. Antibacterial and antiviral drugs are reported as substrates for biological transporters and regarded as exceptions to Lipinski's Ro5^2–4^; nevertheless, these were included in our analyses for setting up parameter boundaries for RO_x_ and examining deviations from Lipinski's Ro5 compliance.

To directly assess impact of physicochemical parameters in RO_x_ on ocular permeability, the relationship between these parameters and *in vitro* permeability (*P*_app_) in corneal and conjunctival tissues of rabbit and porcine was studied for a small subset using semilog plots of data (log *P*_app_) reported by Gukasyan et al.^1^ and Ramsay et al.^8^ (Tables 2 and 3) versus matching RO_x_ parameters (*c*log *D*_pH7.4_, TPSA, and ΔG_o/w_). The correlations between RO_x_ parameters (*c*log *D*_pH7.4_, TPSA, and ΔG_o/w_) and log *P*_app_ indicated that corneal permeability is impacted by the above RO_x_ parameters, while conjunctival permeability is insensitive to the parameters. Notably, permeability experiments for drugs in ocular tissues are often conducted by using dilute solutions of compounds dissolved in aqueous balanced salt buffers at concentrations typically below their saturated solubility, often in the presence of cosolvents, for example, DMSO. While the intrinsic solubility (*S*_int_ or *S*_0_) is considered critical for formulation feasibility of nonionizable drugs, and ionizable drugs exhibit pH-dependet behavior, the overall solubility of molecules in pharmaceutical preparations is relevant in the context of the fraction available to be absorbed from topical ophthalmic doses. Here, actual correlation regression values were poor due to high variability in the literature reported in *in vitro* tissue permeability data.^1,8^ Qualitatively, linear trends showed that RO_x_ parameters have an impact on corneal permeability, whereas conjunctival permeability is not sensitive for the RO_x_ parameters. This can be explained by physiological, mechanistic permeability differences between the conjunctiva and cornea.^14^ Due to the extensive content of this article, the authors decided to report only the summary data for the *in vitro* permeabilities versus RO_x_ parameters (Tables 2 and 3). Tables 2 and 3 examine these correlations by plotting the referenced *in vitro* log *P*_app_ data in rabbit and porcine versus reported RO_x_ parameters in this study.^1,8^

**FIG. 4.**
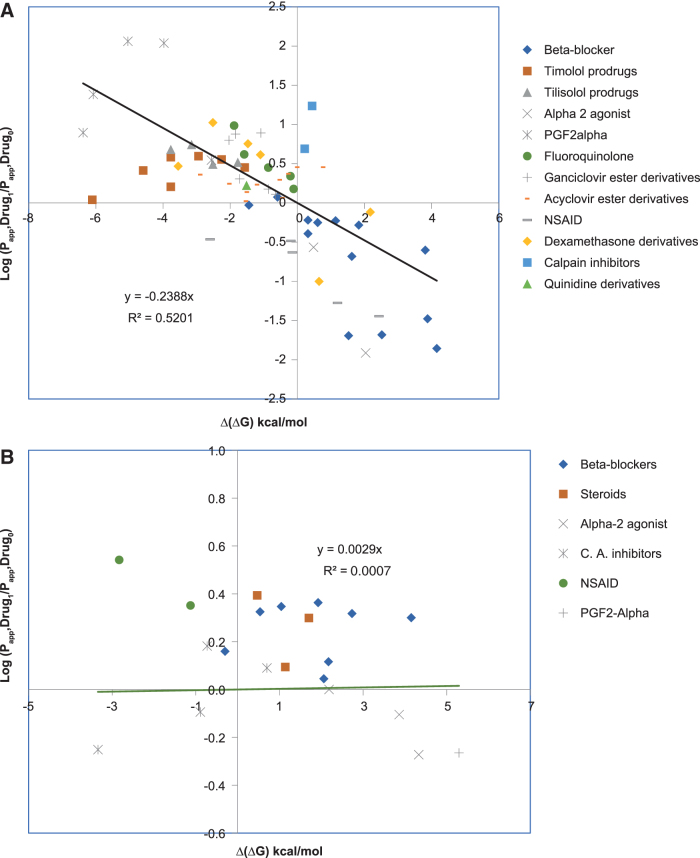
Relationship between changes in the free energy of distribution Δ(ΔG) for 13 congeneric groups of ophthalmic drugs and the *in vitro* corneal **(A)** and conjunctival **(B)** permeability ratios (*P*_app_ optimized drug/*P*_app_ initial drug), assigned as log (*P*_app_ drug_1_/*P*_app_ drug_0_). **(A)** This shows relationship between changes in Δ(ΔG) and corneal permeability ratios. **(B)** This indicates a relationship between changes in Δ(ΔG) and conjunctival permeability ratios. As indicated by (**A**), majority of the congeneric groups (10 of 13) reported by Shirasaki exhibit a lowering of Δ(ΔG) corresponding to a higher corneal permeability ratio, which can be interpreted as an improvement in corneal permeability upon modifications of the molecules. **(B)** This presents Δ(ΔG) for the congeneric groups (*n* = 6) within the 42 ophthalmic drugs with experimental conjunctival *P*_app_^1,8^ that exhibit no impact (poor correlation factor of 0.0029), by the changes in Δ(ΔG) on the conjunctival permeability (see the flat *horizontal line*).

Ocular anatomical, physiological, and biophysical characteristics^1^ for molecular absorption are important considerations in the thermodynamic relationship that indirectly captures physicochemical aspects for predicting epithelial tissue permeability from partition coefficients and free energy of transfer between aqueous and lipid/oil phases, that is, log *D* pH 7.4 and ΔG_o/w_. We adapted thermodynamic models of passive membrane permeability^22,23^ to evaluate their performance against experimental data from isolated tissue-based assays^1,8^ in ocular drug absorption. Based on calculated Δ(ΔG) using Equation (1),^18^ the impact of molecular modifications on ocular permeability changes for congeneric series, for example, prodrugs of an original drug, was predicted. Changes in free energy of transfer [Δ(ΔG)] for 13 topical ophthalmic congeneric groups (total of 67 related molecules) reported by Shirasaki^5^ were calculated, and the results were compared (Fig. 4). Correlations between Δ(ΔG) and experimental corneal permeability ratios (optimized drug/initial drug) are demonstrated. As indicated by Fig. 4A, the majority of congeneric groups (10 of 13) reported in Shirasaki's article exhibits decreasing functions of Δ(ΔG) corresponding to higher corneal permeability ratios, which can be interpreted as an improvement in absorption through this tissue upon incremental modification of listed molecules. The data also suggested that for 3 of 13 congeneric groups (ie, timolol prodrugs, acyclovir ester derivatives, and prostaglandin F2 alpha), the improvement in corneal permeability does not follow the same trendline drawn as the correlation line for all 13 groups. This is most likely due to the nature of functional groups (R) added to modify the initial drug, for example, the ester derivatives of acyclovir. Nevertheless, Fig. 4B, which presents the free energy changes Δ(ΔG) for the congeneric groups (*n* = 6) within the 42 ophthalmic drugs with experimental conjunctival *P*_app_,^1,8^ exhibits no impact, or a very slight effect (correlation factor = 0.0029) of changes in free energy Δ(ΔG) on conjunctival permeability. The above indicates that poor corneal permeability can be mitigated by molecular modification that leads to lowering of free energy of partitioning within congeneric series of drugs.

**FIG. 5.**
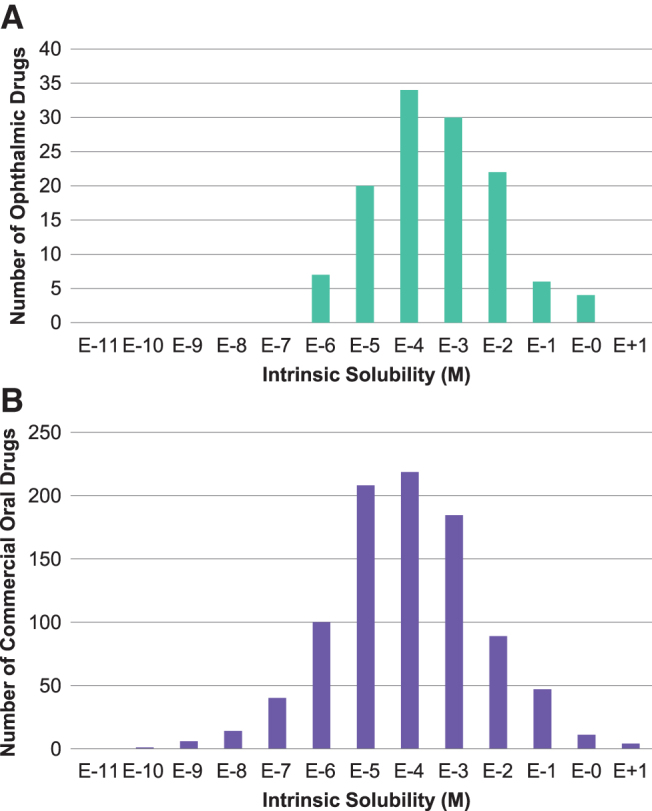
Distributions of intrinsic solubility (*S*_0_) for commercial ophthalmic drugs **(A)** and commercial oral drugs **(B)**, highlighting that the lower limit for solubility of ophthalmic drugs is 1 × 10^−6^ M (1 μM), whereas the lower limit for solubility of oral drugs is shown to be 1 × 10^−10^ M (0.1 nM).

The predicted intrinsic solubility versus the measured solubilities^6,30,31^ of 289 commercial compounds (including ophthalmic drugs)^32–35^ showed a relatively good correlation between the predicted and experimental values (Fig. 3). From Fig. 3, the majority of the compounds (261 of 289) had the calculated solubility within 1 log unit deviation from the experimental values. The standard deviations of the predicted intrinsic aqueous solubility from the experiments are ±0.64 log units for the 289 marketed drugs. A comparative analysis of intrinsic solubility distribution for the commercial ophthalmic drugs versus oral drugs showed higher (absolute concentration) and tighter (more restrictive) solubility margin for the topical ophthalmic products. This emphasizes that drug solubility or solubilization is a critical formulation development attribute for the ophthalmic drugs compared with drugs for oral administration. Figure 5 shows the distributions of intrinsic solubility for ophthalmic drugs (Fig. 5A) ranging from 1 μM to 1 M (4 outliers in Table 1 have experimental solubility above 1 μM), compared with the commercial oral drugs (Fig. 5B) ranging from 0.1 nM to 1 M.

In a foreseeable scenario where a new chemical entity partially satisfies these criteria, in other words is noncompliant with one or more RO_x_ parameter limits, several modifications exemplified in the current dataset can be considered. For ionizable compounds, criteria such as clog *D*_pH7.4_ ≤4, *S*_0_ and *S*_pH7.4_ ≥1 μM should be considered in a physiological context, as acceptable pH range for ophthalmic drug products generally falls within pH 6–8. In addition to formulation optimization strategy discussed earlier, several salt forms (ie, hydrochloride, taratrate, sodium, etc.) also exist in current drug data set, utilizing solubility products of the conjugate acid/base pair for parent drug, or intrinsic buffering capacity within acceptable pH range, to achieve desirable enhancements in final formulated product. Finally, log *P* could be modified by congeneric esterified prodrugs, which can lead to advantageous changes in the free energy of partitioning, partly discussed earlier in context of Shirasaki's review.

To underscore an important detail juxtaposed to the current dataset of successfully developed ophthalmic products is the generally accepted benchmark that ≥90% of a dose from topical eye drops is lost due to the physiological barriers such as rapid tear turnover, nasolacrimal drainage, and reflex blinking. Several product examples listed in the Supplementary Table S1 are designed as suspension, ointment, and emulsion formulations, which are accepted pharmaceutical approaches partially responsible for reducing effects of mentioned elimination mechanisms by virtue of increased precorneal residence time and durable maintenance of a drug-saturated tear film. Our proposed RO_x_ parameters scrutinize molecular thermodynamics of solution and diffusion within the context of ocular physiology and anatomy; therefore, their direct impact on anatomical barriers requires additional research considering discrete formulation-related factors.

## Conclusions

The predevelopment screening of new ophthalmic drug candidates, whether they currently exist or are newly designed chemical entities, is resource intensive, and therefore *in silico* assessment of their developability based on successfully commercialized physicochemical design space could result in a cost-effective approach. Results of this study outline 4 thermodynamic physicochemical parameters beyond the descriptors in literature (ie, Lipinski's Ro5), and include only successfully developed topical ophthalmic products for derivation: ΔG_o/w_, TPSA, *c*log *D*_pH7.4_, and solubility (*S*_0_ and *S*_pH7.4_). While the physicochemical evaluation presented in this study suggests that molecular descriptors defined by Lipinski's Ro5 (MW, log *P*, nHBD, and nHBA) may not be complete measures for assessing the “drugability” of topical ophthalmic drug candidates, new limits on ΔG_o/w_, TPSA, *c*log *D*_pH7.4_, and solubility (*S*_0_ and *S*_pH7.4_) are recommended. Based on our results from parameter distribution analysis of 145 approved ophthalmic drugs, outcomes of this study propose the following parameter limits defined as Rule of Thumb for Ophthalmics (RO_x_): *c*log *D*_pH7.4_ ≤4.0, TPSA ≤250 Å^2^, *Δ*G_o/w_ ≤20 kJ/mol, and solubility (*S*_0_ and *S*_pH7.4_) ≥1 μM, which can be used for developability assessment of new topical ophthalmic drug candidates.

## Supplementary Material

Supplemental data

## Data Sharing Statement

Available data are reported in this article.
