# Supplementary material for: Eyes on Lipinski's Rule of Five: A New “Rule of Thumb” for Physicochemical Design Space of Ophthalmic Drugs
Source: J Ocul Pharmacol Ther. 2022 Jan 28;38(1):43–55. doi: 10.1089/jop.2021.0069 (PMC8817695; doi:10.1089/jop.2021.0069)
Supplement: Supplemental data [file Supp_Table1.docx]

**Table 1.** Summary of Physicochemical Properties of Approved Ophthalmic Drugs^*^ Used for this Study

| **Drug’s Name** | **(Parameters in RO_x_) ^**^** | | | | **(Parameters in Ro5) ^**^** | | | |
| --- | --- | --- | --- | --- | --- | --- | --- | --- |
|  | ***c*log D** “_pH 7.4_” | **TPSA**  (Å^2^) | **c S_(pH 7.4)_**  (M) | **ΔG^***^**  (kJ/mol) | **MW**  (Da) | **nHBD** | **nHBA** | ***c* log P** |
| Acebutolol | -0.38 | 87.7 | 9.3E-01 | -9.97 | 336.43 | 3 | 6 | 1.68 |
| Acetazolamide | -0.68 | 151.7 | 1.8E-02 | 1.84 | 222.25 | 3 | 7 | -0.31 |
| Acetylcholin chloride | -3.50 | 26.3 | 6.0E-01 | **20.77** | 146.21 | 0 | 3 | -3.5 |
| Acyclovir | -1.23 | 114.8 | 8.9E-03 | 7.30 | 225.20 | 4 | 8 | -1.23 |
| Alcaftadine | 1.69 | 38.1 | 1.9E-02 | -16.98 | 307.39 | 0 | 4 | 2.86 |
| Ampicillin | -1.86 | 138.0 | 2.8E-02 | -5.58 | 349.41 | 4 | 7 | 0.94 |
| Antazoline | 1.09 | 27.6 | 2.1E-01 | -21.31 | 265.35 | 1 | 3 | 3.59 |
| Apraclonidine | -0.41 | 62.4 | 6.6E-03 | -8.19 | 245.11 | 4 | 4 | 1.38 |
| Atenolol | -1.82 | 84.6 | 2.5E+00 | -1.42 | 266.34 | 4 | 5 | 0.24 |
| Atropine | -0.35 | 49.8 | 9.1E-01 | -11.04 | 289.37 | 1 | 4 | 1.86 |
| Azelastine HCl | 2.14 | 35.9 | 3.2E-04 | -25.52 | 381.9 | 0 | 4 | 4.30 |
| Azithromycin | 0.15 | 180.1 | 1.0E+00 | -19.53 | **748.98** | 5 | **14** | 3.29 |
| Aztreonam | -6.21 | 238.2 | 5.9E+00 | 7.18 | 435.44 | 5 | **13** | -1.21 |
| Bacitracin | -5.77 | **556.2** | 1.0E-05 | 18.76 | **1,422.69** | **20** | **33** | -3.16 |
| Benoxinate | 2.30 | 64.8 | 1.5E-02 | -22.20 | 308.42 | 2 | 5 | 3.74 |
| Bepotastine | 0.84 | 62.7 | 1.7E-03 | -19.88 | 388.89 | 1 | 5 | 3.35 |
| Besifloxacin | -0.80 | 86.9 | 1.5E-05 | -7.66 | 393.84 | 3 | 6 | 1.29 |
| Betamethasone | 1.92 | 94.8 | 1.7E-04 | -11.40 | 392.46 | 3 | 5 | 1.92 |
| Betaxolol HCl | 0.81 | 50.7 | 5.2E-01 | -17.04 | 307.43 | 2 | 4 | 2.87 |
| Bilastine | 2.46 | 67.6 | 4.9E-04 | -29.44 | 463.61 | 1 | 6 | 4.96 |
| Bimatoprost | 2.37 | 89.8 | 8.7E-04 | -14.07 | 415.57 | 4 | 5 | 2.37 |
| Brilliant Blue G | -0.38 | 153.0 | **4.0E-08** | 5.82 | **833.05** | 3 | 10 | -0.98 |
| Brimonidine | -1.97 | 62.2 | 3.5E-01 | -5.64 | 292.13 | 2 | 5 | 0.95 |
| Brimonidine (tartrate) | -1.68 | 62.2 | 6.0E-01 | -7.36 | 292.13 | 2 | 5 | 1.24 |
| Brinzolamide | -0.15 | 163.8 | 3.2E-03 | 0.06 | 383.51 | 3 | 8 | -0.01 |
| Bromfenac sodium | -0.1 | 80.4 | 4.1E-01 | -18.22 | 356.15 | 3 | 4 | 3.07 |
| Bufuralol | 1.42 | 45.4 | 4.1E-02 | -21.31 | 261.36 | 2 | 3 | 3.59 |
| Bunazosin | 1.22 | 93.8 | 2.8E-04 | -9.20 | 373.45 | 2 | 8 | 1.55 |
| Carbinoxamine | 1.35 | 25.4 | 4.6E-03 | -15.73 | 290.79 | 0 | 3 | 2.65 |
| Carteolol | -0.30 | 70.6 | 1.2E+00 | -10.92 | 292.37 | 3 | 5 | 1.84 |
| Cephalexin | -3.15 | 138.0 | 6.3E-02 | 2.08 | 347.39 | 4 | 7 | -0.35 |
| Cetirizine | -0.55 | 53.0 | 2.4E-02 | -14.96 | 388.89 | 1 | 5 | 2.52 |
| Chloramphenicol | 1.02 | 115.4 | 8.9E-03 | -6.05 | 323.13 | 3 | 7 | 1.02 |
| Chlorotetracycline | -2.43 | 182.6 | 1.4E-03 | -3.86 | 478.88 | **7** | 10 | 0.65 |
| Ciprofloxacin | -2.60 | 72.9 | 6.8E-04 | 1.78 | 331.34 | 2 | 6 | -0.30 |
| Clindamycin | 1.78 | 127.6 | 2.6E-02 | -12.58 | 424.98 | 4 | 7 | 2.12 |
| Clonidine | 1.18 | 36.4 | 6.2E-04 | -10.68 | 230.09 | 2 | 3 | 1.80 |
| Cromolyn sodium | -2.95 | 165.9 | 2.1E+00 | -10.68 | 468.37 | 3 | **11** | 1.80 |
| Cyclopentolate | 1.55 | 49.8 | 2.5E-02 | -14.07 | 291.39 | 1 | 4 | 2.37 |
| Cyclosporine | 1.80 | **278.8** | 9.8E-06 | -10.68 | 1,202.61 | 5 | **23** | 1.80 |
| Cysteamine | -2.37 | 64.8 | 5.6E+00 | 0.18 | 77.15 | 2 | 1 | -0.03 |
| Dapiprazole | 2.35 | 37.2 | 6.5E-03 | -16.86 | 325.45 | 0 | 5 | 2.84 |
| Demecarium | -3.14 | 59.1 | 1.1E-05 | 18.64 | **556.78** | 0 | 8 | -3.14 |
| Dexamethasone | 1.92 | 94.8 | 1.7E-04 | -11.40 | 392.46 | 3 | 5 | 1.92 |
| Dexamethasone acetate | 2.82 | 100.9 | 6.9E-05 | -16.74 | 434.50 | 2 | 6 | 2.82 |
| Diclofenac | 1.47 | 49.3 | 2.2E-02 | -26.59 | 296.15 | 2 | 3 | 4.48 |
| Difluprednate | 3.18 | 107.0 | 3.5E-05 | -18.88 | 508.55 | 1 | 7 | 3.18 |
| Dipivefrin | 0.64 | 84.9 | 3.1E-01 | -14.07 | 351.44 | 2 | 6 | 2.37 |
| Diquafosol Tetrasodium | -15.48 | **432.8** | 7.1E+00 | **50.33** | **790.31** | **10** | **27** | -8.48 |
| Dorzolamide HCl | -1.19 | 151.3 | 3.4E-02 | 1.31 | 324.44 | 3 | 6 | -0.22 |
| Echothiophate | -2.21 | 70.6 | 1.1E-01 | 13.12 | 256.32 | 0 | 4 | -2.21 |
| Emedastine | 0.22 | 33.5 | 1.5E-01 | -16.03 | 302.41 | 0 | 5 | 2.70 |
| Epinastine HCl | 1.17 | 41.6 | 3.5E-03 | -16.44 | 249.31 | 2 | 3 | 2.77 |
| Erythromycin | 1.69 | 193.9 | 1.3E-02 | -14.48 | **733.93** | 5 | **14** | 2.44 |
| Ethoxzolamide | 1.75 | 118.9 | 3.9E-04 | -10.92 | 258.32 | 2 | 5 | 1.84 |
| Fluconazole | 0.70 | 81.7 | 6.9E-03 | -4.15 | 306.27 | 1 | 7 | 0.70 |
| Fluorescein | 3.55 | 76.0 | 6.9E-05 | -21.13 | 332.31 | 2 | 5 | 3.56 |
| Fluorometholone | 2.45 | 74.6 | 4.6E-05 | -14.54 | 376.46 | 2 | 4 | 2.45 |
| Fluorometholone acetate | 2.64 | 80.7 | 3.5E-05 | -15.67 | 418.50 | 1 | 5 | 2.64 |
| Flurbiprofen | 0.61 | 37.3 | 9.5E-02 | -22.67 | 244.26 | 1 | 2 | 3.82 |
| Ganciclovir | -1.72 | 135.0 | 1.1E-02 | 10.21 | 255.23 | 5 | 9 | -1.72 |
| Gatifloxacin | -2.05 | 82.1 | 4.3E-04 | -1.13 | 375.39 | 2 | 7 | 0.19 |
| Gentamicin | -7.90 | 199.7 | 2.1E+00 | 12.76 | 477.60 | **11** | **12** | -2.15 |
| Gramicidin D | **5.54** | **519.9** | **2.5E-12** | -32.88 | **1,811.22** | **20** | **35** | **5.54** |
| Hydrocortisone | 1.66 | 94.8 | 4.4E-04 | -9.85 | 362.46 | 3 | 5 | 1.66 |
| Hydroxyamphetamine | -1.03 | 46.3 | 1.4E+00 | -5.82 | 151.21 | 3 | 2 | 0.98 |
| Idoxuridine | -0.95 | 99.1 | 2.6E-02 | 4.63 | 354.10 | 3 | 7 | -0.78 |
| Indomethacin | 1.14 | 68.5 | 1.7E-02 | -23.86 | 357.79 | 1 | 5 | 4.02 |
| Isopropyl unoprostone | **4.79** | 83.8 | 2.5E-05 | -28.43 | 424.61 | 2 | 5 | 4.79 |
| Ketorolac | -0.92 | 59.3 | 1.4E+00 | -15.31 | 255.27 | 1 | 4 | 2.58 |
| Ketotifen | 2.84 | 48.6 | 2.5E-03 | -24.10 | 309.43 | 0 | 2 | 4.06 |
| Latanoprost | **4.11** | 87.0 | 2.3E-04 | -24.40 | 432.59 | 3 | 5 | 4.11 |
| Latanoprost acid | 0.26 | 98.0 | 3.2E-01 | -16.32 | 390.51 | 4 | 5 | 2.75 |
| Latanoprostene bunod | **4.25** | 145.1 | 3.3E-05 | -25.23 | **507.62** | 3 | 9 | 4.25 |
| Levobetaxolol | 0.81 | 50.7 | 5.2E-01 | -17.04 | 307.43 | 2 | 4 | 2.87 |
| Levobunolol | 0.44 | 58.6 | 8.7E-01 | -15.25 | 291.39 | 2 | 4 | 2.57 |
| Levocabastine (HCl) | 1.98 | 64.3 | 1.3E-04 | -26.59 | 420.52 | 1 | 4 | 4.48 |
| Levofloxacin | -2.00 | 73.3 | 7.9E-04 | -1.01 | 361.37 | 1 | 7 | 0.17 |
| Lidocaine (gel) | 1.71 | 32.3 | 6.0E-02 | -13.83 | 234.34 | 1 | 3 | 2.33 |
| Lifitegrast | -0.73 | 142.4 | 3.5E-04 | -19.05 | **615.48** | 2 | 9 | 3.21 |
| Lincomycin | 0.27 | 147.8 | 3.0E-01 | -3.74 | 406.54 | 5 | 8 | 0.63 |
| Liothyronine | **4.07** | 92.8 | 2.9E-06 | -24.45 | **650.97** | 4 | 5 | 4.12 |
| Lodoxamide | -4.50 | 156.6 | 3.2E+00 | -1.48 | 311.63 | 4 | 9 | 0.25 |
| Lomefloxacin | -2.70 | 72.9 | 1.5E-03 | 2.26 | 351.35 | 2 | 6 | -0.38 |
| Loratadine | **5.32** | 42.4 | 2.5E-06 | -31.58 | 382.88 | 0 | 4 | **5.32** |
| Lornoxicam | 0.27 | 136.2 | 4.0E-03 | -13.83 | 371.82 | 2 | 7 | 2.33 |
| Loteprednol etabonate | 3.54 | 99.1 | 3.9E-06 | -21.01 | 466.95 | 1 | 7 | 3.54 |
| Medrysone | 3.08 | 54.4 | 9.3E-05 | -18.28 | 344.49 | 1 | 3 | 3.08 |
| Mepyramine | 1.62 | 28.6 | 5.0E-02 | -18.10 | 285.38 | 0 | 4 | 3.05 |
| Methazolamide | -0.03 | 138.9 | 4.0E-02 | -1.90 | 236.27 | 2 | 7 | 0.32 |
| Methotrexate | -5.42 | 210.5 | 8.9E-02 | 3.32 | 454.44 | **7** | **13** | -0.56 |
| Methylprednisolone | 1.97 | 94.8 | 3.7E-04 | -11.69 | 374.47 | 3 | 5 | 1.97 |
| Metipranolol | 0.47 | 67.8 | 1.1E+00 | -14.96 | 309.4 | 2 | 5 | 2.52 |
| Moxifloxacin | -1.47 | 82.1 | 5.8E-05 | -4.63 | 401.43 | 2 | 7 | 0.78 |
| Nadolol | -0.90 | 82.0 | 1.9E+00 | -7.36 | 309.4 | 4 | 5 | 1.24 |
| Naphazoline | 0.49 | 24.4 | 3.6E-01 | -19.53 | 210.27 | 1 | 2 | 3.29 |
| Natamycin | -2.92 | 231.0 | 7.9E-03 | 2.37 | **665.73** | **8** | **14** | -0.40 |
| Nedocromil | -2.80 | 121.2 | 1.5E+00 | -13.06 | 371.34 | 2 | 8 | 2.20 |
| Neomycin | -9.15 | **353.1** | 7.1E+00 | **28.97** | **614.64** | **19** | **19** | -4.88 |
| Nepafenac | 1.38 | 86.2 | 1.4E-03 | -8.19 | 254.28 | 4 | 4 | 1.38 |
| Netarsudil | 2.78 | 94.3 | 3.5E-06 | -19.77 | 453.53 | 3 | 6 | 3.33 |
| Norfloxacin | -2.74 | 72.9 | 2.4E-03 | 3.38 | 319.33 | 2 | 6 | -0.57 |
| Ofloxacin | -2.08 | 73.3 | 1.7E-03 | -1.01 | 361.37 | 1 | 7 | 0.17 |
| Olopatadine | 1.53 | 49.8 | 1.0E-03 | -23.98 | 337.41 | 1 | 4 | 4.04 |
| Oxymetazoline | 0.86 | 44.6 | 2.8E-01 | -21.55 | 260.37 | 2 | 3 | 3.63 |
| Oxytetracycline | -4.25 | 201.9 | 1.3E-02 | 7.84 | 460.43 | **8** | **11** | -1.32 |
| Pazufloxacin mesylate | -1.48 | 92.9 | 3.5E-04 | -2.73 | 318.30 | 3 | 6 | 0.46 |
| Pemirolast | -1.78 | 87.1 | 2.2E-01 | -1.19 | 228.21 | 1 | 7 | 0.20 |
| Pheniramine | 0.98 | 16.1 | 4.7E-01 | -16.44 | 240.34 | 0 | 2 | 2.77 |
| Phenylephrine | -2.03 | 52.5 | 6.0E+00 | 0.83 | 167.21 | 3 | 3 | -0.14 |
| Pilocarpine | 0.23 | 44.1 | 4.0E-02 | -2.31 | 208.26 | 0 | 4 | 0.39 |
| Pindolol | -0.20 | 57.3 | 8.9E-01 | -11.04 | 248.32 | 3 | 4 | 1.86 |
| Polymyxin B | -11.16 | **490.7** | 8.3E-01 | **25.05** | **1,203.48** | **23** | **29** | -4.22 |
| Povidone-iodine | 0.38 | 20.3 | 1.5E+00 | -2.26 | 111.14 | 0 | 2 | 0.38 |
| Prednisolone acetate | 2.33 | 100.9 | 1.7E-04 | -13.83 | 402.48 | 2 | 6 | 2.33 |
| Prednisolone Na phosphate | -4.88 | 151.2 | 4.0E+00 | 0.06 | 440.42 | 4 | 8 | -0.01 |
| Proparacaine | 1.84 | 64.8 | 2.6E-02 | -19.41 | 294.39 | 2 | 5 | 3.27 |
| Propranolol | 1.20 | 41.5 | 3.8E-01 | -19.35 | 259.34 | 2 | 3 | 3.26 |
| Proxodolol | -0.85 | 98.9 | 1.5E+00 | -7.72 | 351.40 | 2 | 8 | 1.30 |
| Pyrilamine | 1.09 | 28.6 | 1.7E-01 | -18.10 | 285.38 | 0 | 4 | 3.05 |
| Quinidine | 1.82 | 45.6 | 1.2E-02 | -17.93 | 324.42 | 1 | 4 | 3.02 |
| Rebamipide | -1.48 | 95.5 | 1.5E-01 | -13.77 | 370.79 | 3 | 6 | 2.32 |
| Riboflavin (Vitamin B2) | -1.14 | 155.1 | 8.5E-05 | 6.77 | 376.36 | 5 | 10 | -1.14 |
| Rimexolone | 3.80 | 54.4 | 1.0E-05 | -22.56 | 370.52 | 1 | 3 | 3.80 |
| Ripasudil | 0.24 | 70.7 | 1.4E-02 | -11.22 | 323.39 | 1 | 5 | 1.89 |
| Sulfacetamide | -2.73 | 97.6 | 1.3E+00 | 3.62 | 214.24 | 3 | 5 | -0.61 |
| Suprofen | -0.29 | 82.6 | 5.8E-01 | -17.81 | 260.31 | 1 | 3 | 3.00 |
| Tacrolimus | **4.10** | 178.4 | 5.1E-06 | -24.34 | **804.02** | 3 | **13** | 4.1 |
| Tafluprost | **4.24** | 76.0 | 5.2E-05 | -25.17 | 452.53 | 2 | 5 | 4.24 |
| Testosterone | 3.16 | 37.3 | 7.2E-05 | -18.76 | 288.42 | 1 | 2 | 3.16 |
| Tetracaine | 2.26 | 41.6 | 2.8E-02 | -19.59 | 264.36 | 1 | 4 | 3.30 |
| Tetracycline | -3.55 | 181.6 | 2.9E-03 | 4.51 | 444.43 | 7 | 10 | -0.76 |
| Timolol | -0.79 | 108.0 | 3.6E+00 | -9.08 | 316.42 | 2 | 7 | 1.53 |
| Tizanidine | -1.63 | 90.4 | 8.3E-01 | -7.66 | 253.71 | 2 | 5 | 1.29 |
| Tobramycin | -7.22 | **268.2** | 7.1E+00 | **24.51** | 467.51 | **15** | **14** | -4.13 |
| Travoprost | 3.98 | 96.2 | 3.4E-05 | -23.62 | **500.55** | 3 | 6 | 3.98 |
| Trifluridine | -0.62 | 99.1 | 3.7E-02 | 2.67 | 296.20 | 3 | 7 | -0.45 |
| Trimethoprim | 0.96 | 105.5 | 4.0E-03 | -6.65 | 290.32 | 4 | 7 | 1.12 |
| Trypan Blue | -9.56 | **392.9** | 1.5E-02 | 18.16 | **872.88** | **10** | **20** | -3.06 |
| Tropicamide | 1.54 | 53.4 | 3.0E-03 | -9.20 | 284.35 | 1 | 4 | 1.55 |
| Unoprostone | 1.02 | 94.8 | 4.0E-02 | -21.55 | 382.53 | 3 | 5 | 3.63 |
| Vitamin E | **10.3** | 29.5 | **8.3E-09** | -61.14 | 430.71 | 1 | 2 | **10.3** |
| Vizomitin | **4.23** | 34.1 | **5.6E-10** | -25.11 | **537.69** | 0 | 2 | 4.23 |
| Voriconazole | 1.39 | 76.7 | 1.2E-03 | -8.25 | 349.31 | 1 | 6 | 1.39 |
| Vidarabine | -0.98 | 139.5 | 6.0E-03 | 5.82 | 267.24 | 5 | 9 | -0.98 |

**^*^**The ophthalmic drugs examined for physicochemical descriptors in Ro5 and RO_x_ include antibacterial drugs, which were exempt from Lipinski’s Ro5. We included antibacterial and antiviral drugs (i.e., gatifloxacin, gentamicin, azithromycin, idoxuridine, levofloxacin, sulfacetamide, natamycin, norfloxacin, ganciclovir, gramicidin, moxifloxacin, trimethoprim, tobramycin, neomycin, chlorotetracycline, and trifluridine) in our parameter distribution analysis to examine these drugs for parameter boundaries in RO_x_. The drug properties that are outliers for the RO_x_ and deviate from the Ro5 are highlighted in **bold**.

^**^Definition of ROx and RO5 parameters: log D_pH7.4_, distribution coefficient at physiological pH; log P, partition coefficient; MW, molecular weight; nHBA, number of H-bond acceptors; nHBD, number of H-bond donors; TPSA, topological polar surface area; Ro5, rule of 5; RO_x_, “Rule of Thumb” for Ophthalmics; S_pH7.4_, solubility at physiological pH; ΔG_o/w_, free energy of partitioning.

**^***^**ΔG (free energy of distribution/partitioning) was calculated by using lop P for non-ionizable drugs, while for ionizable drugs the ΔG was calculated by using log D at pH 7.4 (pH of the tear film) because partition coefficient of the ionizable drugs at pH7.4 is relevant for ocular drug delivery.
